# Brain-penetrating and lesion-triggered hydrogen sulfide nanocarrier for multiple pathogenic interventions in Parkinson's disease therapy

**DOI:** 10.1016/j.mtbio.2026.103806

**Published:** 2026-11-03

**Authors:** Jia Ke, Shiyi Yu, Hong Yuan, Fuqiang Hu

**Affiliations:** a Department of Pharmacy, Affiliated Hospital of Zunyi Medical University, Zunyi, 563000, China; b College of Pharmaceutical Science, Zhejiang University, Hangzhou, 310058, China; c National Key Laboratory of Advanced Drug Delivery and Release Systems, Zhejiang University, Hangzhou, 310058, China

**Keywords:** Parkinson's disease, H_2_S nanocarrier, Brain-penetrating, Stimulus response, Multifunctional therapy

## Abstract

The defective hydrogen sulfide (H_2_S) metabolism in brain has emerged as a crucial pathogenic event and therapeutic target in Parkinson's disease (PD). However, current H_2_S delivery approaches for PD remain predominantly limited by the blood-brain barrier (BBB) and on-demand supply. This work aimed to upgrade the delivery of H_2_S to across BBB, thereby adequately harnessing its neuroprotective effect in PD brain. We developed a brain-penetrating, ROS-responsive H₂S nanocarrier (RVG29/TK@Lip/GYY) for targeted H₂S delivery in the PD brain. In this system, the H₂S donor GYY4137 was encapsulated within the liposomal core, and a ROS-responsive thioketal (TK) linker was incorporated into the lipid bilayer to enable on-demand H₂S release in the oxidative PD microenvironment. The liposomal surface was modified with the brain-homing peptide RVG29 to facilitate BBB crossing and neuronal targeting. This formulation achieved a good BBB penetration rate of 22.12%. In addition, pathological evaluation demonstrated that the controlled H_2_S release by RVG29/TK@Lip/GYY exerted multiple PD pathogenic interventions, including scavenging excessive ROS, alleviating nitrative stress, decreasing neuronal apoptosis, suppressing glial cell activation, and promoting nerve regeneration. Importantly, RVG29/TK@Lip/GYY treatment significantly attenuated the motor deficits of PD model mice. Collectively, this study not only provides a promising H_2_S nanocarrier for PD multiple pathological therapy, but also contributes to the development of H_2_S delivery in other neurodegenerative diseases treatment.

## Graphical abstract

**Figure:**
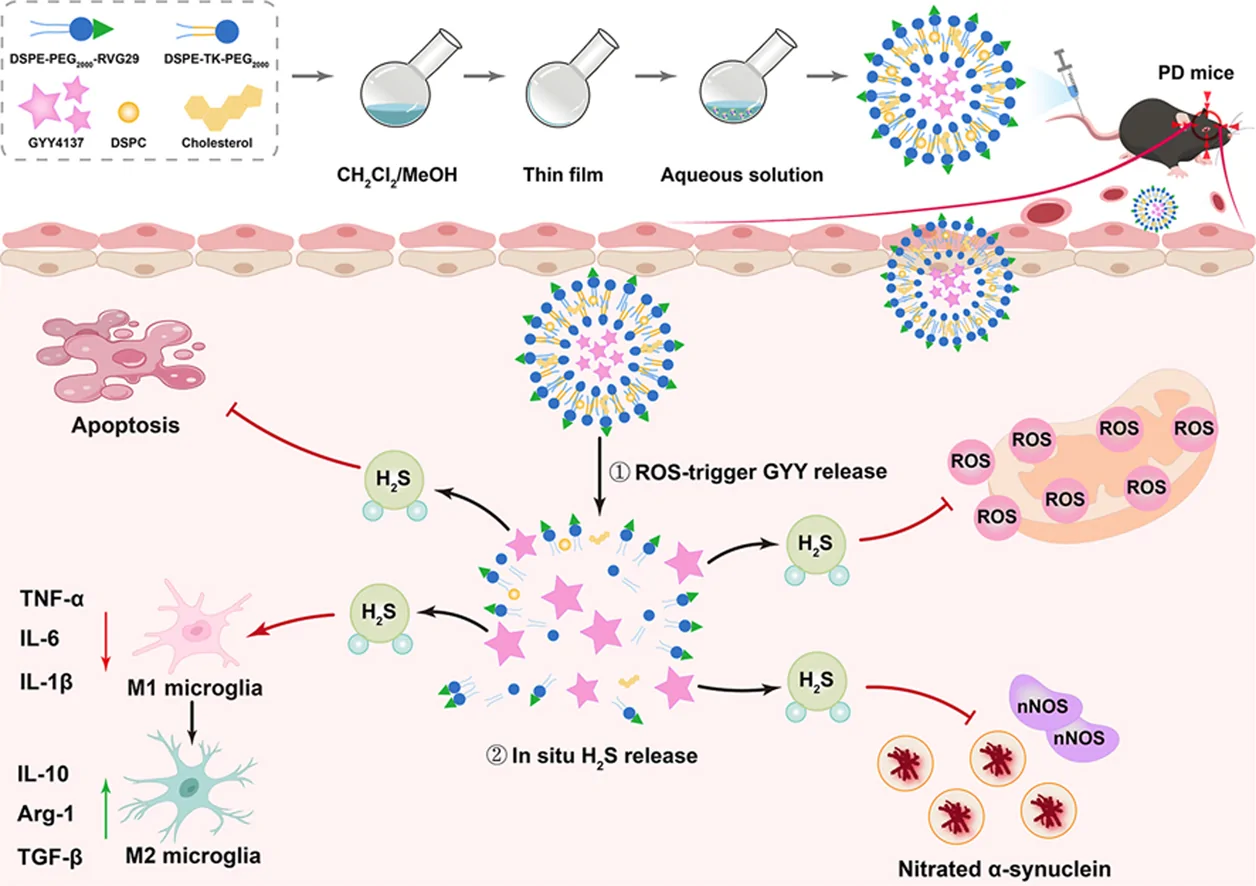

## 1 Introduction

Parkinson's disease (PD) is a chronic progressive neurodegenerative disorder that characterized by the loss of dopaminergic neurons in the substantia nigra (SN), and clinically manifested as motor symptom deficits [1], [2]. Due to its multifactorial and intricate pathogenesis, no effective clinical therapies have been developed to date [3]. In addition, current pharmacological strategies mainly centered on the regulation of dopamine (DA) metabolism, which was failed to address the underlying etiologies, also accompanied with severe side effects [3], [4]. Nowadays, emerging evidence suggested that PD pathogenesis was tightly associated with various pathological factors such as oxidative/nitrative stress, neuroinflammation responses, microglial homeostasis and accumulation of neuronal α-synuclein (α-syn) [5], [6], [7], [8], [9], [10]. Furthermore, these pathogenetic factors drive and magnify with a vicious circle process, eventually resulting in dopaminergic neuron degeneration in PD [11], [12]. Therefore, a versatile therapeutic approach that around above causative and manifold PD pathogenesis holds great potential for intensive PD treatment.

As a vital gaseous signaling molecule, hydrogen sulfide (H_2_S) has been attracted increasing attention in pathological improvements and therapy, due to its anti-oxidative stress [13], anti-inflammatory [14], [15], and anti-apoptosis [16]. Moreover, mechanistic studies reveal that declining endogenous brain H_2_S levels are closely associated with impaired sulfhydration-dependent Parkin activation, defective clearance of neurotoxic proteins, and α-syn accumulation in PD brain [17], [18], [19], [20], suggesting that restoring H_2_S signaling may simultaneously correct multiple PD-related pathologies. Small-molecule inorganic H_2_S donors such as sodium sulfide and sodium hydrosulfide generally suffered from short half-lives, systemic diffusion, and local high concentration toxic effects, hindering the *in vivo* application [21]. While organic H_2_S donors like GYY4137 can stable release H_2_S, their poor brain permeability and limited lesion selectivity mostly impeded brain delivery [22]. To date, various H_2_S delivery systems have been reported, including responsive hydrogels, metal-organic frameworks, and nanomotors [23], [24], [25], [26]. However, critical limitations still persist. Many of these systems suffer from local or invasive administration, insufficient brain enrichment and neuron-specific targeting, non-specific carrier degradation, and brain biocompatibility caused by metal components [27], [28]. Moreover, the integrity and highly restricted permeability of blood-brain barrier (BBB) significantly impedes the efficient delivery of therapeutic agents, which require a high dosage or long-term administration [29], [30]. In this case, a desirable H_2_S delivery nanocarrier that integrates BBB-penetrating and PD pathological microenvironment response to achieve the multiple curative effects of H_2_S is highly urgent, yet still scarce. RVG29 is a 29-amino acid peptide derived from the rabies virus glycoprotein, which has been reported to endow nanomaterials with BBB penetrability via binding to the nicotinic acetylcholine receptor (nAChR) on neurons and brain capillary endothelial cells [31], [32], [33]. On the other hand, the elevated ROS in PD pathology aroused us the design of microenvironment-triggered release [9], [34]. Nevertheless, the feasible construction of RVG29-based H_2_S nanocarrier which can be triggered by ROS in PD brain is still a considerable challenge.

Herein, we presented a brain-targeted and ROS-triggered H_2_S nanocarrier termed as RVG29/TK@Lip/GYY. As illustrated in Scheme 1, the modification of RVG29 can significantly enhance BBB penetrative ability, the elevated ROS in PD pathology triggered the degradation of thioketal linker (TK) and then released H_2_S donor (GYY4137), thus achieving the on-demand release of H_2_S in PD brain. *In vitro* results revealed that the controlled release of H_2_S from RVG29/TK@Lip/GYY exerted various neuroprotective effects in PD model nerve cells, including reducing oxidative and nitrative stress, alleviating apoptosis, and inhibiting neuronal nitric oxide synthase. In addition, RVG29/TK@Lip/GYY effectively relieved neuroinflammation, restored microglial homeostasis and recovered dopaminergic neurons damage *in vivo*. Notably, behavioral experiment results demonstrated that the treatment of RVG29/TK@Lip/GYY significantly improved the motor function of PD model mice. Overall, this nanotherapeutic strategy for controlled H_2_S release offers a promising strategy for PD multifunctional treatment.

**Scheme 1:**
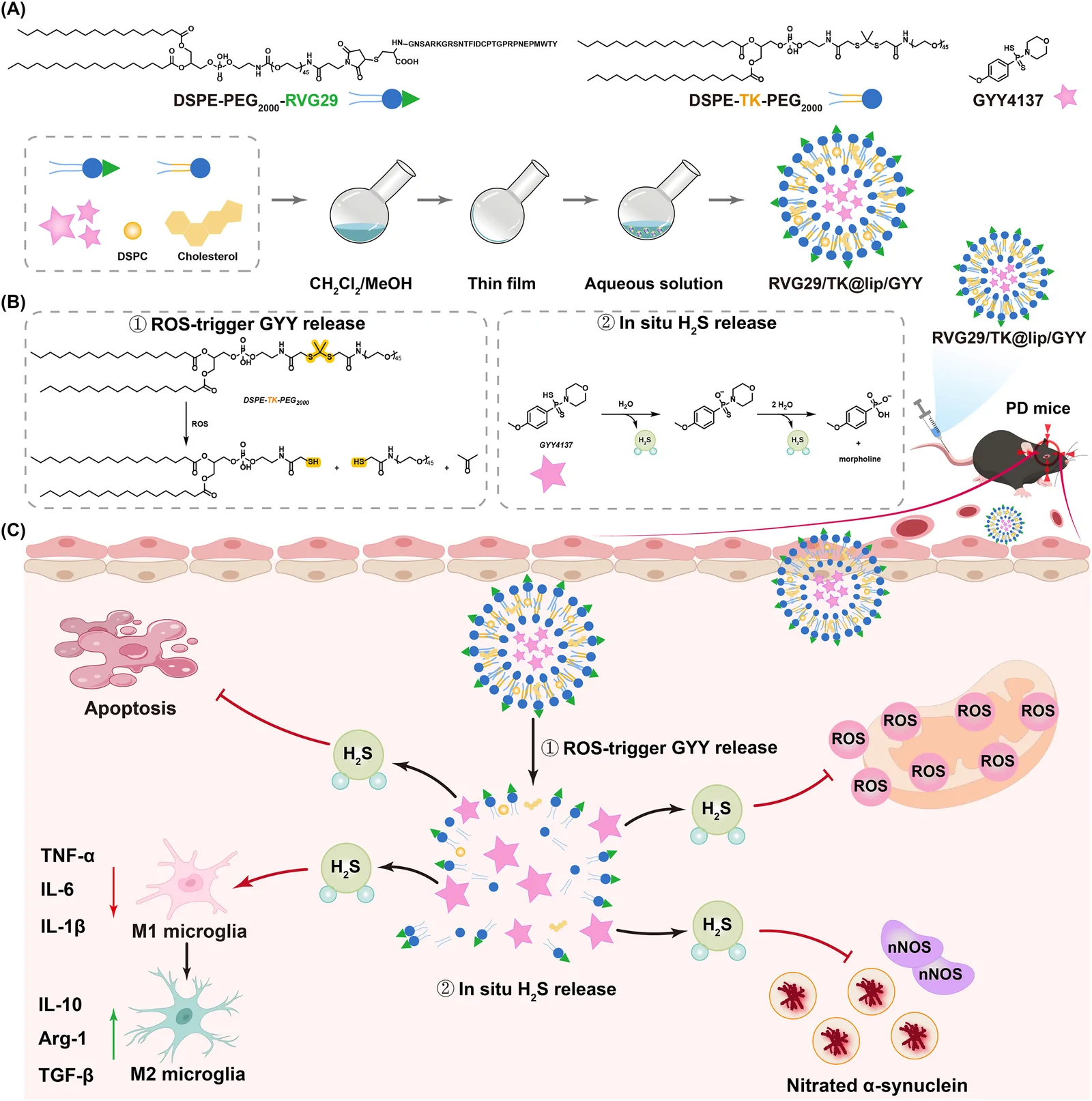
(A) Chemical structure of components and the preparation of RVG29/TK@Lip/GYY. (B) The mechanism of DSPE-TK-PEG_2000_ towards ROS and the release of H_2_S by GYY4137. (C) Schematic illustration of RVG29/TK@Lip/GYY therapeutic mechanisms in PD focal brain.

## 2 Results and discussion

### 2.1 Synthesis and characterization of RVG29/TK@Lip/GYY

Firstly, DSPE-PEG_2000_-RVG29 was synthesized according to our previous work [35], and fully confirmed by ^1^H NMR (Fig. S1). Then, the GYY4137 loaded ROS-responsive nanocarrier (RVG29/TK@Lip/GYY) and the nanocarrier without GYY4137 (RVG29/TK@Lip) were prepared by the film emulsion method, respectively (Fig. 1A). As depicted in Fig. 1B, transmission electron microscopy (TEM) illustrated that both RVG29/TK@Lip/GYY and Lip/GYY showed spherical vesicles with bilayer lipid structures. Additionally, the mean hydrodynamic diameter of RVG29/TK@Lip/GYY and Lip/GYY were 107.7 ± 3.0 nm and 115.3 ± 4.7 nm, respectively. Besides, both of them showed a negative zeta potential −22.9 ± 2.3 mV and −40.0 ± 0.7 mV, respectively (Fig. 1B–E). The encapsulation efficiency (EE%) and drug loading (DL) proportion of GYY in RVG29/TK@Lip/GYY and Lip/GYY were calculated as 57.3 ± 2.8% and 53.6 ± 2.1%, 4.6 ± 0.2% and 4.3 ± 0.2%, respectively (Fig. 1F). Meanwhile, the stability of RVG29/TK@Lip/GYY was evaluated by monitoring changes in particle size and polydispersity index (PDI) in PBS containing 10% Foetal Bovine Serum (FBS) over 5 days. As shown in Fig. S2, the particle size difference remained within 9 nm throughout the incubation period, while the PDI showed only a slight increase from 0.20 ± 0.04 to 0.23 ± 0.01, indicating the good stability of RVG29/TK@Lip/GYY under physiological condition.

**Fig. 1:**
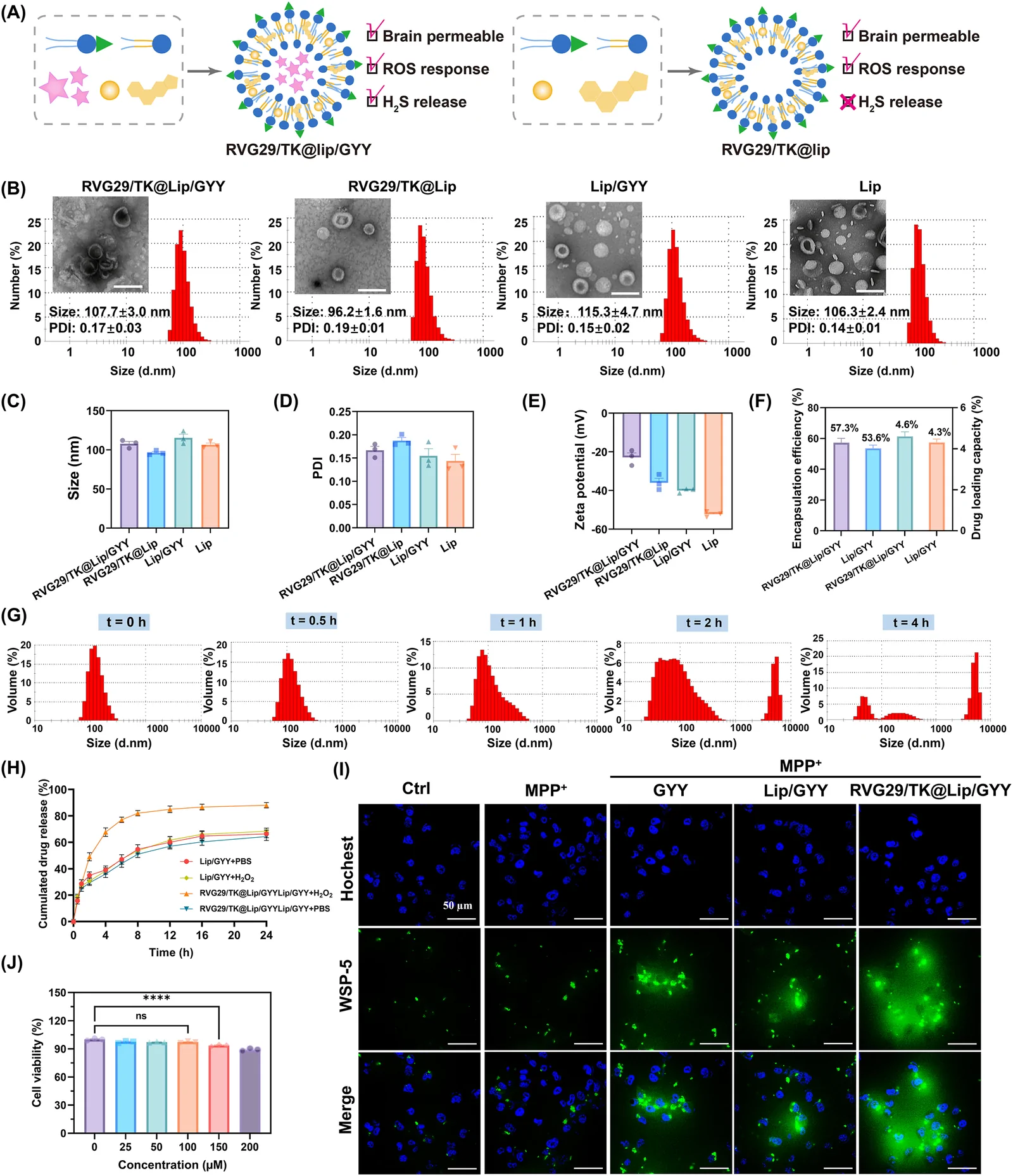
Characterization and ROS responsiveness of RVG29/TK@Lip/GYY. (A) The preparation process of RVG29/TK@Lip/GYY and RVG29/TK@Lip, and their properties comparison. (B) The TEM images and number-weighted size distribution of RVG29/TK@Lip/GYY, RVG29/TK@Lip, Lip/GYY and Lip; scale bar = 200 nm. (C) Particle sizes, (D) PDI and (E) zeta potential of RVG29/TK@Lip/GYY, RVG29/TK@Lip, Lip/GYY and Lip, respectively. (F) The EE% and DL% of GYY in RVG29/TK@Lip/GYY and Lip/GYY. (G) ROS-sensitive behavior of RVG29/TK@Lip/GYY. (H) GYY release profile of RVG29/TK@Lip/GYY and Lip/GYY under different H_2_O_2_ concentration (0 μM and 100 μM). (I) H_2_S release profile of SH-SY5Y cells after different intervention. (J) The cell viability of SH-SY5Y cells after treatment with RVG29/TK@Lip/GYY for 24 h. Results are reported as means ± SEM (*n* = 3); one-way ANOVA; TuKey's multiple comparisons test; *p < 0.05, **p < 0.01, ***p < 0.001, ****p < 0.0001 and ns, not significant.

### 2.2 ROS-responsive properties of RVG29/TK@Lip/GYY

Next, we investigated the ROS-responsive release capacity of RVG29/TK@Lip/GYY under H_2_O_2_ (100 μM). The volume-weighted particle size distribution was measured at different time points. As displayed in Fig. 1G and S3, compared with Lip/GYY, the initial peak (133.0 nm) of RVG29/TK@Lip/GYY significantly decreased, the smaller particle size (49 nm) and the larger particle size (5220 nm) obviously increased in volume-weighted size distribution of at 4 h (Table S1). Moreover, the released content of GYY4137 was measured to evaluate the ROS-sensitive release efficiency. As presented in Fig. 1H, the GYY4137 release from RVG29/TK@Lip/GYY was reached 84.24% at 8 h in the presence of H_2_O_2_ (100 μM), which was higher than that of 51.1% under normal conditions. These results suggested the excellent ROS-sensitive behavior of the TK linker in RVG29/TK@Lip/GYY (Fig. S4). Subsequently, the intracellular H_2_S release performance of RVG29/TK@Lip/GYY was investigated by Washington State Probe-5 (WSP-5), a specific fluorescent probe for H_2_S. As depicted in Fig. 1I, a faint fluorescence was observed in the MPP^+^-treated group, indicating the low cellular level of H_2_S in PD microenvironment. However, SH-SY5Y cells displayed an increased fluorescence signal upon the treatment of RVG29/TK@Lip/GYY in PD model cells, suggesting that RVG29/TK@Lip/GYY processes a superior ROS-responsive for precise temporal and spatial releasing H_2_S from GYY4317 in PD focal cells (Fig. S5). To advance the application of RVG29/TK@Lip/GYY in living cells and PD brain, cytocompatibility was evaluated using a standard MTT assay. As shown in Fig. 1J, SH-SY5Y cells maintained 89.3% viability following 24-h treatment with 200 μM RVG29/TK@Lip/GYY. In addition, other components of RVG29/TK@Lip/GYY, including the GYY4137 and Lip/GYY, also exhibited excellent biocompatibility (Fig. S6), implying the promising application of RVG29/TK@Lip/GYY in PD brain therapy research.

### 2.3 Cellular uptake and BBB penetration of RVG29/TK@Lip/GYY

We subsequently evaluated the cellular uptake efficiency and the BBB permeability of RVG29/TK@Lip/GYY *in vitro*. For investigating cellular uptake, both RVG29/TK@Lip and Lip were labeled with DiI dye, the cellular uptake was performed by confocal laser scanning microscope (CLSM) and flow cytometry (FCM). As illustrated in Fig. 2A and B, both the RVG29/TK@Lip/DiI and Lip/DiI exhibited a time-dependent uptake efficiency both in bEnd.3 and SH-SY5Y cells, and the intracellular DiI fluorescence of RVG29/TK@Lip/DiI group was consistently stronger than that of Lip/DiI group throughout the entire internalization process. As displayed in Fig. 2C and D, after 4 h incubation, RVG29/TK@Lip/DiI demonstrated significantly enhanced cell internalization rates than Lip/DiI in both cell lines: 57.60% vs. 36.67% in bEnd.3 cells, and 64.78% vs. 27.13% in SH-SY5Y cells. These results suggested that the modification of RVG29 in H_2_S nanocarrier could efficiently enhance the cellular uptake efficiency. Then, the BBB permeability was assessed using an *in vitro* BBB model (Fig. 2E). As seen in Fig. 2F, RVG29/TK@Lip/DiR possessed the more prominent transport capability (22.12%) than that of Lip/DiR (6.19%), suggesting the feasibility of RVG29 for the enhancement of BBB penetration. Taken together, these results indicated that RVG29/TK@Lip/GYY can effectively traverse BBB and enter nerve cells for further exerting its H_2_S releasing function in PD brain.

**Fig. 2:**
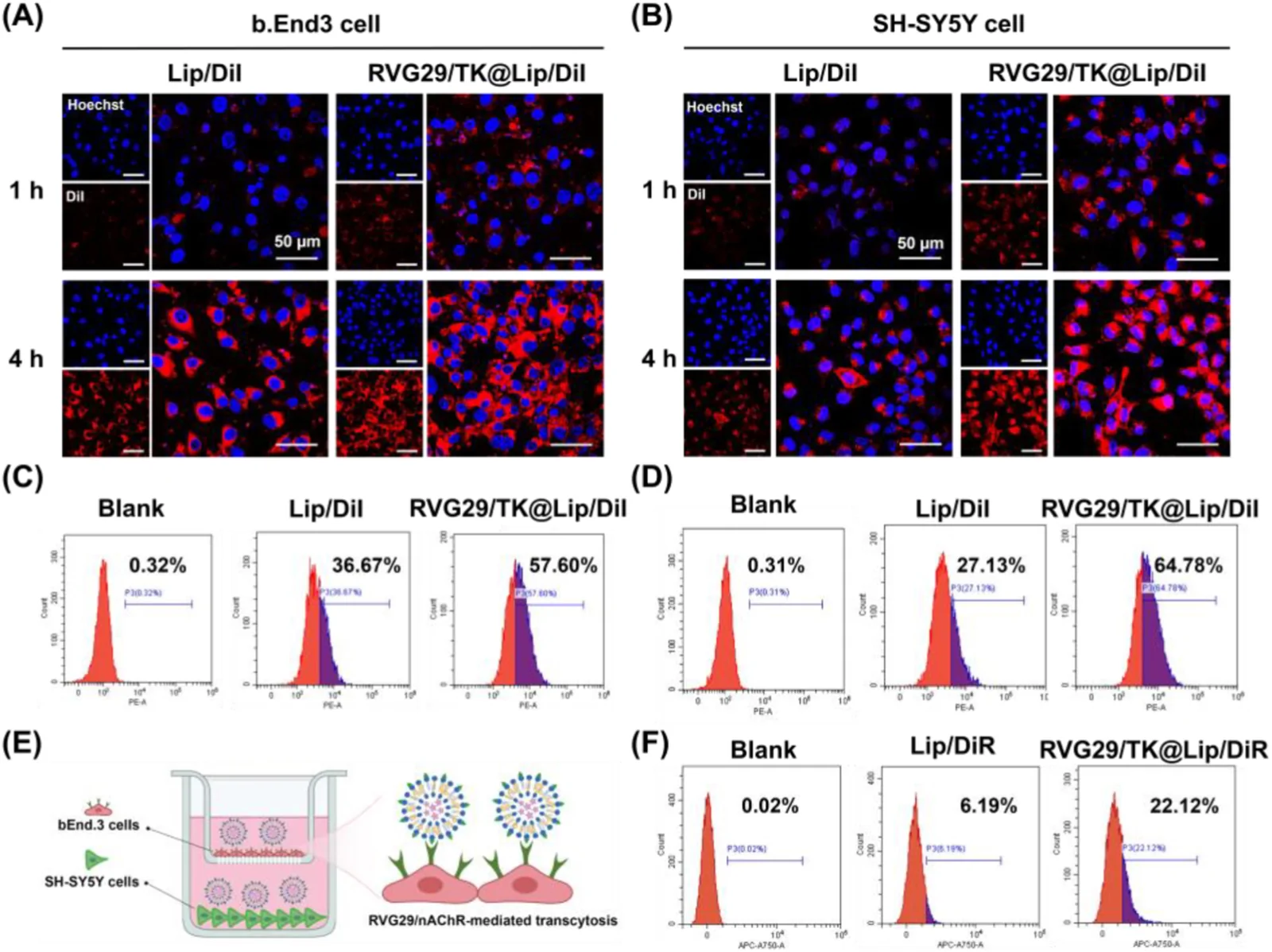
Cellular uptake and *in vitro* BBB transportation of RVG29/TK@Lip/GYY. Cellular uptake of DiI-labeled drug-delivery system in (A) bEnd.3 cells and (B) SH-SY5Y cells at 1 and 4 h was observed by CLSM; scale bar = 50 μm. Quantitative evaluation of the cellular uptake based on FCM of (C) bEnd.3 cells and (D) SH-SY5Y cells at 4 h. (E) Schematic illustration of the transport of nanoparticles across *in vitro* BBB cell model. (F) The transport capability of RVG29/TK@Lip/DiR and Lip/DiR. Results are reported as means ± SEM (*n* = 3); one-way ANOVA; TuKey's multiple comparisons test; *p < 0.05, **p < 0.01, ***p < 0.001, ***p < 0.0001 and ns, not significant.

### 2.4 In vitro therapeutic evaluation of RVG29/TK@Lip/GYY

Oxidative stress plays a critical role in driving neuronal apoptosis and dopamine depletion in PD progression, while H_2_S possesses potential ROS scavenging activity [36], [37]. We next investigated whether RVG29/TK@Lip/GYY could attenuate oxidative stress in the PD model SH-SY5Y cells. To this end, intracellular ROS levels were assessed using the ROS-sensitive fluorescent probe DCFH-DA. As shown in Fig. 3A, compared with Ctrl group, SH-SY5Y cells incubated with MPP^+^ exhibited a significant increase in green fluorescence intensity, indicative of elevated ROS production in this PD model cells. Remarkably, compared with the MPP^+^ group and Lip/GYY group, cellular fluorescence intensity in the RVG29/TK@Lip/GYY treatment group was reduced by approximately 36.88% and 21.80% (Fig. 3B), respectively, indicating that the controlled release of H_2_S from RVG29/TK@Lip/GYY can effectively eliminate excess intra-neuronal ROS in PD microenvironment.

**Fig. 3:**
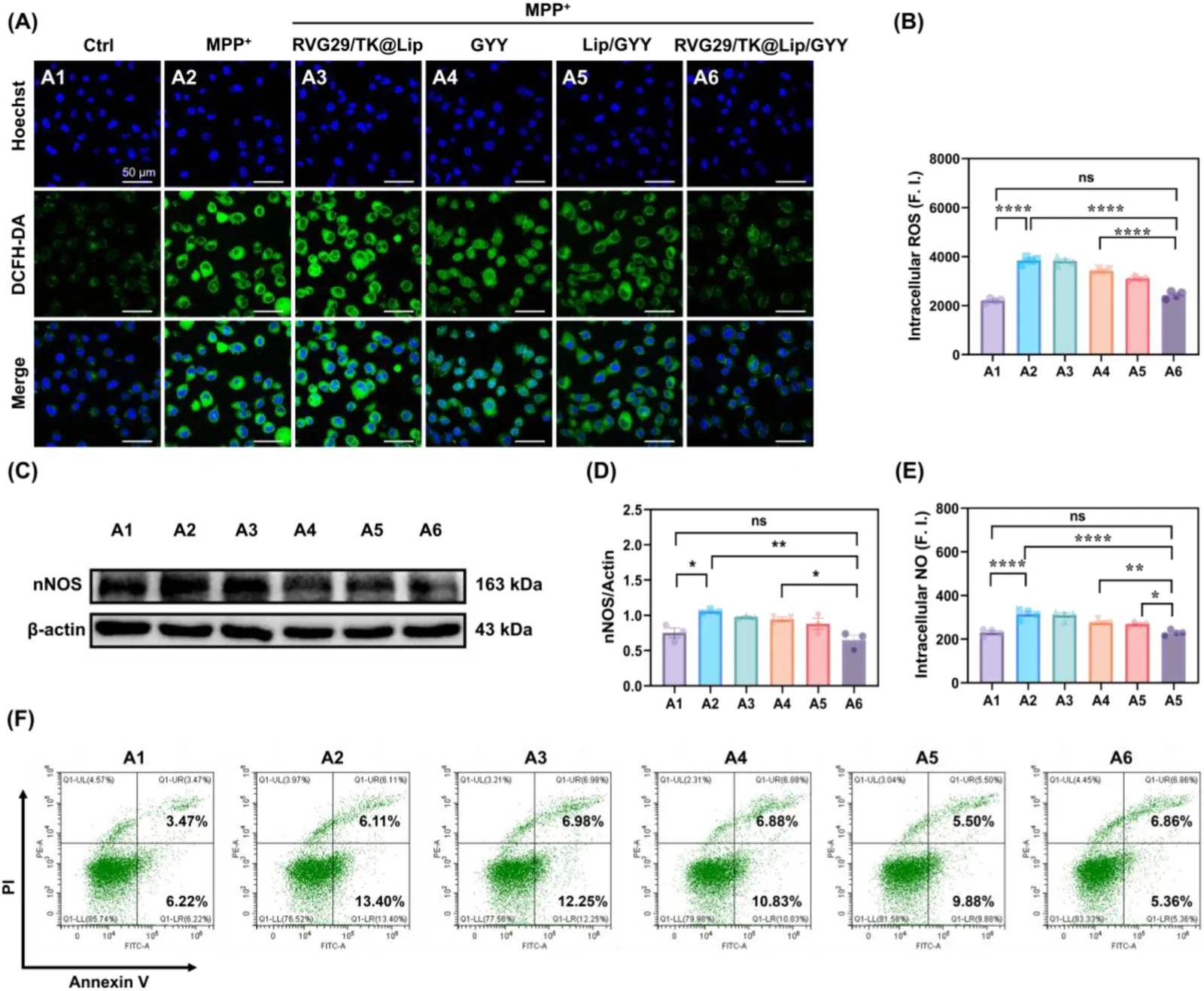
*In vitro* reducing oxidative stress and possessing neuroprotective abilities of RVG29/TK@Lip/GYY. (A) The ROS levels of SH-SY5Y cells was detected by DCFH-DA fluorescent probe; scale bar = 50 μm. (B) The intracellular ROS content based on fluorescence intensity quantification of each group SH-SY5Y cells in (A), *n* = 4. (C) WB analysis of the nNOS protein expression in each group SH-SY5Y cells in (A). (D) Semi-quantitative results of the relative expression of nNOS in (C). (E) Intracellular NO content based on fluorescence intensity quantification of each group in (A). (F) Apoptosis assay of SH-SY5Y cells after different treatment conditions in (A). Results are reported as means ± SEM (*n* = 3); one-way ANOVA; TuKey's multiple comparisons test; *p < 0.05, **p < 0.01, ***p < 0.001, ****p < 0.0001 and ns, not significant.

Emerging evidence indicates that nitrosative stress in neuronal cells is critically linked to the pathological accumulation of reactive nitrogen species (RNS), primarily driven by the upregulation and hyperactivity of neuronal nitric oxide synthase (nNOS) [38], [39]. This nNOS-mediated RNS overproduction has been implicated in *α*-syn misfolding, dopaminergic neuron loss, and Lewy body formation [40], [41]. Thus, the level of nNOS was determined in various group cells. As displayed in Fig. 3C, the incubation of MPP^+^ could markedly cause an elevation expression of nNOS compared with the Ctrl group. On the contrary, when the treatment with RVG29/TK@Lip/GYY, the expression of nNOS in SH-SY5Y cells was appreciably decreased more than other groups (Fig. 3D). In addition, the level of cellular nitric oxide (NO) was also analyzed by using DAF-FM-DA, a NO specific fluorescent probe. As depicted in Fig. 3E, the fluorescence intensity in the MPP^+^ group increased by 37.0% compared to the Ctrl group. In contrast, the fluorescence intensity in the RVG29/TK@Lip/GYY group, Lip/GYY group, and GYY group decreased by 27.6%, 14.6% and 12.6% relative to the MPP^+^ group, respectively. These findings indicated that RVG29/TK@Lip/GYY significantly reduces intracellular NO production via inhibiting the activity of nNOS. Afterwards, the anti-apoptosis capacity of RVG29/TK@Lip/GYY was further investigated. As seen in Fig. 3F, the FCM results demonstrated that the RVG29/TK@Lip/GYY treatment group exhibited a marked reduction of total apoptosis ratio (12.2%, average values in Fig. S7) compared to the MPP^+^-treated group (19.5%), GYY group (17.0%) and Lip/GYY group (15.4%). In addition, the results of MTT assays in each group suggested the crosscurrent, which further indicating the significant feature of microenvironment-response for H_2_S release of RVG29/TK@Lip/GYY in PD cells (Fig. S8). Consequently, these results highlighted the controlled release of H_2_S by RVG29/TK@Lip/GYY in mitigating oxidative and nitrative stress as well as diminishing apoptosis in PD microenvironment.

### 2.5 RVG29/TK@Lip/GYY improves motor function in PD mice

Guided by above *in vitro* therapeutic efficiency, we seek to evaluate the potential therapeutic ability of RVG29/TK@Lip/GYY in PD mice. Before this, the biodistribution profiling and brain-targeting efficiency were investigated by the DiR-labeled RVG29/TK@Lip (Fig. 4A). As presented in Fig. 4B and S9, the fluorescence signal reached maximum after injection 4 h. Group with RVG29/TK@Lip/DiR displayed a 1.75-folds fluorescence increase than that of Lip/DiR, indicating the incremental brain accumulation of RVG29/TK@Lip/DiR than non-targeted Lip/DiR. In addition, brain tissues in RVG29/TK@Lip/DiR treatment group also showed enhanced fluorescence signal than Lip/DiR group (Fig. S10). After that, we systematically evaluated the therapeutic efficacy of RVG29/TK@Lip/GYY in MPTP-induced PD model. Following the animal experimental protocol (Fig. 4C), the motor function of mice in the six groups (G1: Ctrl, G2: PD model, G3: PD model + RVG29/TK@Lip, G4: PD model + GYY, G5: PD model + Lip/GYY, and G6: PD model + RVG29/TK@Lip/GYY) was evaluated by a series of behavioral assessments. As the open-field tests in Fig. 4D, significant motor deficits were observed in the PD model mice, including shorter total movement distance (8.7 m), reduced distance in central zone entries (0.6 m), slower speed (0.03 m/s), and increased immobile time (167.3 s) compared with Ctrl mice (30.5 m, 5.5 m, 0.10 m/s and 23.2 s). Meanwhile, both PD model + RVG29/TK@Lip, PD model + GYY and PD model + Lip/GYY showed slight amelioration, which due to the lack of H_2_S release in PD model + RVG29/TK@Lip, the low brain-targeting and H_2_S delivery efficiency in PD model + GYY and non-responsive governed release in PD model + Lip/GYY, respectively. Conversely, the treatment of RVG29/TK@Lip/GYY significantly improved these motor impairments of PD model mice with longer total movement distance (27.6 m), increased distance in central zone entries (4.1 m), enlarged speed (0.09 m/s), and lessened immobile time (26.5 s) (Fig. 4E–I). Moreover, the pole test was applied to estimate the motor and coordination abilities of PD mice (Fig. 4J). As exhibited in Fig. 4K, the total descendent time of PD model mice to the bottom was approximately 20.1 s which was much longer than the Ctrl mice (4.7 s). After the treatment with RVG29/TK@Lip, GYY, Lip/GYY, the motion coordination ability could be faintly improved. Notably, the descendent time of PD model + RVG29/TK@Lip/GYY was significantly decreased to 8.4 s which was closer to the Ctrl mice. In addition, the rotarod test (Fig. 4L and M) showed that the retention time of PD model mice was 39.2 s, which was obviously lower than the Ctrl group (138.8 s). However, the PD model mice treated with RVG29/TK@Lip/GYY were enough to be capable of remaining on the rotarod for extended periods (112.0 s) in comparison with RVG29/TK@Lip (41.3 s), GYY (68.5 s) and Lip/GYY (70.0 s). Taken together, these findings suggested that RVG29/TK@Lip/GYY effectively mitigates motor function deficits in PD model mice and exerts its potential therapeutic application for PD.

**Fig. 4:**
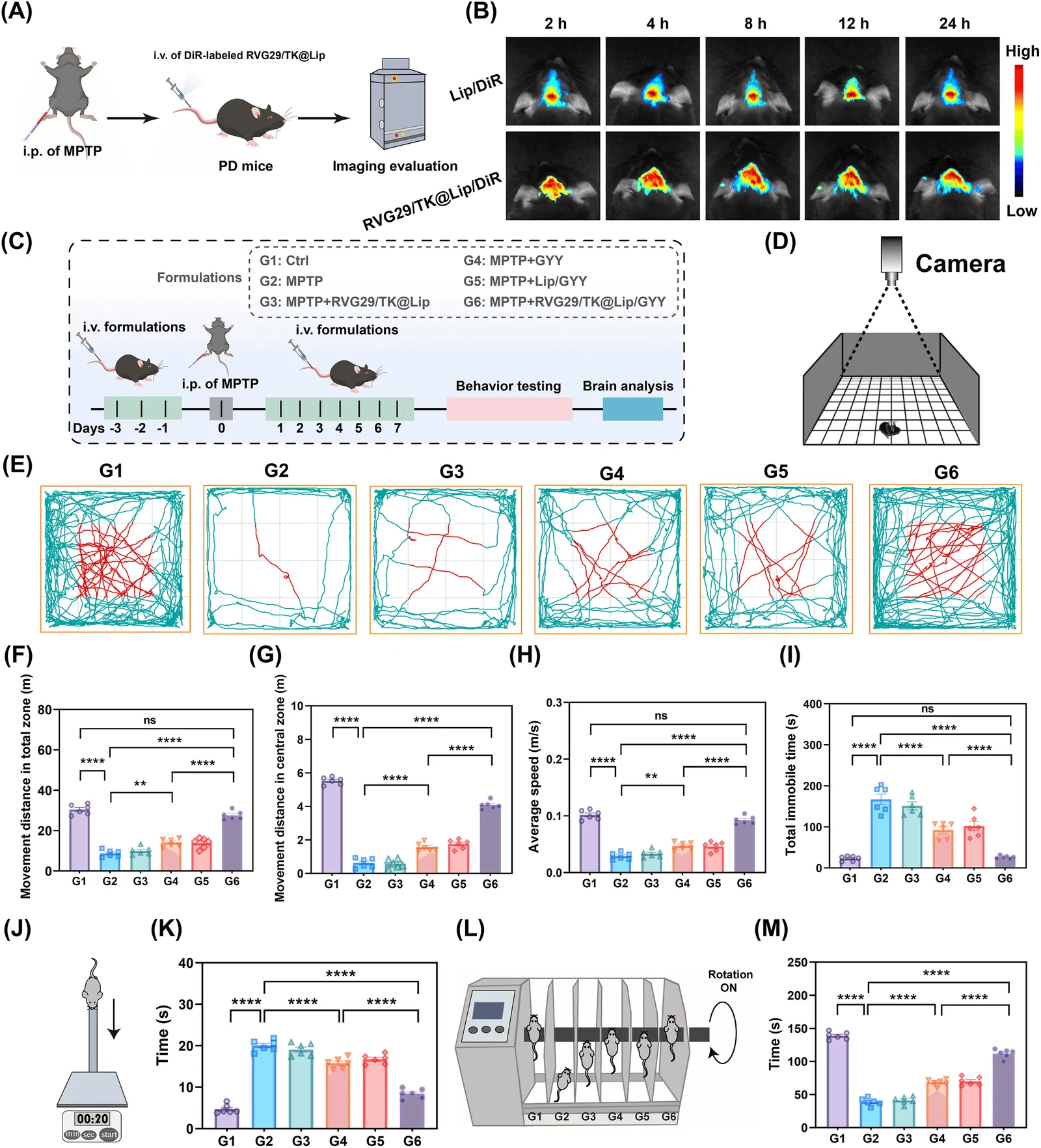
Therapeutic evaluations of RVG29/TK@Lip/GYY in MPTP-induced PD mice. (A) Schematic illustration of *in vivo* imaging protocol. (B) *In vivo* fluorescence images of different formulations at different time (*n* = 3). (C) Experimental design overview. (D) Schematic diagram of the open-field test. (E) Representative behavioral routes of healthy and MPTP-treated mice after different formulations administration. Open-field test indexes: (F) movement total distance, (G) central area distance, (H) averaged speed, and (I) total immobile time. (J) Schematic diagram of the pole test. (K) Total descend time. (L) Schematic diagram of the rotarod test. (M) Total latency time in the rotarod test. Results are reported as means ± SEM (*n* = 6); one-way ANOVA; TuKey's multiple comparisons test; *p < 0.05, **p < 0.01, ***p < 0.001, ****p < 0.0001 and ns, not significant.

### 2.6 RVG29/TK@Lip/GYY rescues dopaminergic neurons

To verify the adaptive H_2_S release from RVG29/TK@Lip/GYY and its therapeutic efficacy in PD model mice. We firstly examined H_2_S levels in the striatum (ST), a key region affected in PD pathology, using a commercial detection kit (Fig. 5A). As presented in Fig. 5B, H_2_S levels in PD model mice were markedly lower than those in the Ctrl group. Treatment with the free H_2_S donor GYY4137 or the non-brain-targeting Lip/GYY produced only a faint increase in H_2_S levels. In contrast, the RVG29/TK@Lip/GYY group showed a substantial elevation in H_2_S levels, confirming the effective brain delivery of H_2_S by RVG29/TK@Lip/GYY. Subsequently, we evaluated the protective effect of RVG29/TK@Lip/GYY on dopaminergic neurons by immunohistochemical (IHC) staining of tyrosine hydroxylase (TH) in the ST and substantia nigra pars compacta (SNpc). As depicted in Fig. 5C, TH-positive neurons in both regions were dramatically decreased in the PD model group, while RVG29/TK@Lip/GYY treatment largely restored TH expression compared with other treatment groups. In addition, WB results in Fig. 5D revealed that TH protein expression in RVG29/TK@Lip/GYY group was significantly upregulated, which increased 1.12-fold (SN) and 1.34-fold (ST) compared with the severe DA loss in PD model mice, respectively (Fig. S11). Meanwhile, the DA content in the SN and ST of each group was also measured by ELISA assay. As illustrated in Fig. 5E and F, treatment with RVG29/TK@Lip/GYY exerted an evident DA supplement effect in the PD model brain, which correlated positively with TH levels. Therefore, these results demonstrated that RVG29/TK@Lip/GYY evidently ameliorated DA deficiency and protected dopaminergic neurons in PD brain.

**Fig. 5:**
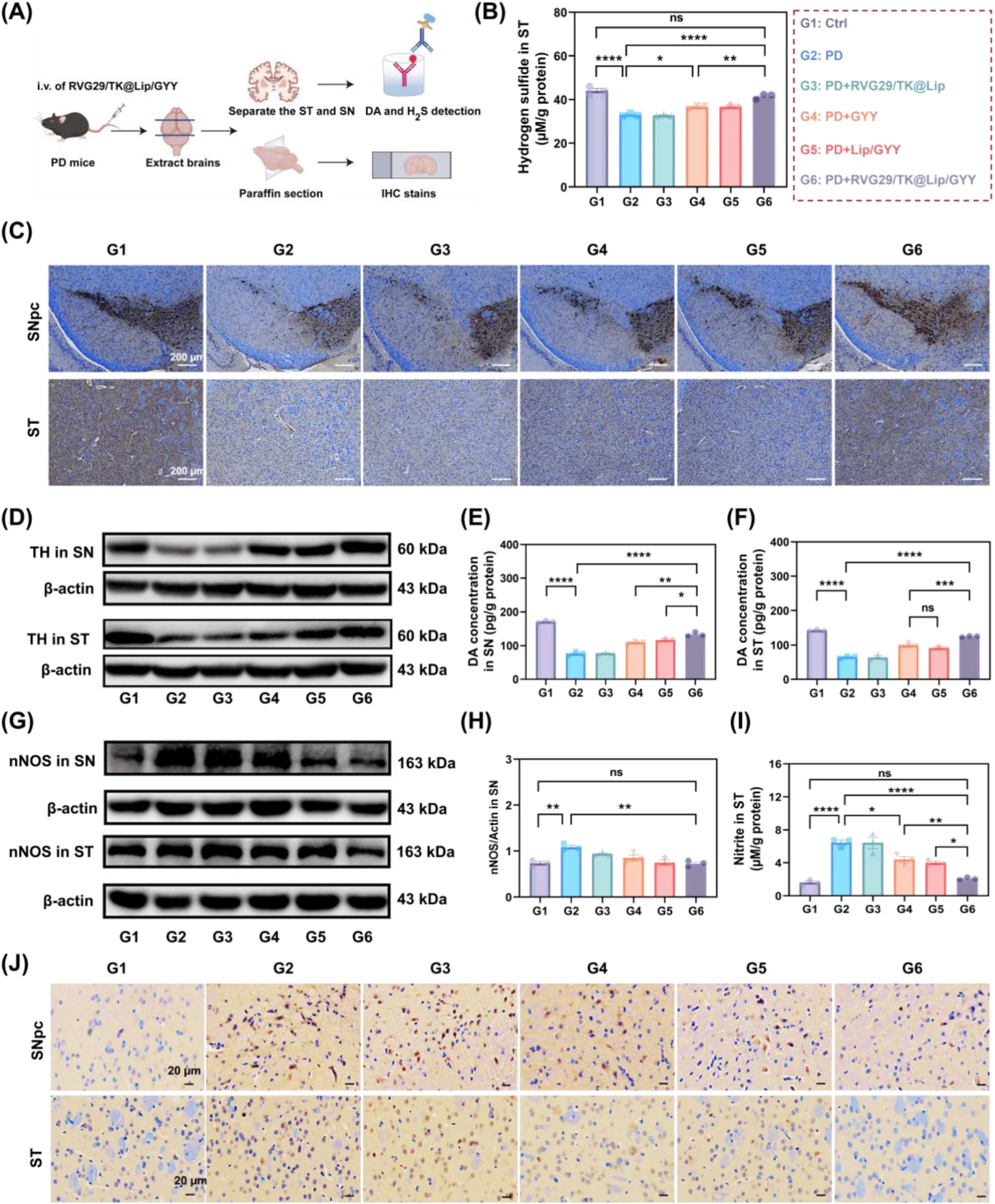
The effect of RVG29/TK@Lip/GYY on recovering dopaminergic neurons and anti-nitrification stress. (A) Schematic diagram of the DA and H_2_S content determination, IHC stains procedure. (B) The H_2_S content in the ST after different formulations administration. (C) Representative IHC images of TH in the SNpc and ST; scale bar = 200 μm. (D) WB analysis of the TH expression level in the SN and ST. The DA content in the (E) SN and (F) ST after different formulations administration. (G) WB analysis of the nNOS expression level in the SN and ST. (H) The relative semi-quantitative results of nNOS in (G). (I) The nitrite content in the ST after different formulations administration. (J) IHC stains results of the expression of Nitro-*α*-syn; scale bar = 20 μm. Results are reported as means ± SEM (*n* = 3); one-way ANOVA; TuKey's multiple comparisons test; *p < 0.05, **p < 0.01, ***p < 0.001, ****p < 0.0001 and ns, not significant.

### 2.7 RVG29/TK@Lip/GYY mitigates nitrification stress

The elevated levels of nitrite, nitrate, and nitrated α-synuclein (Nitro-α-syn) was closely associated with the serious nitrative stress in PD brain [42], [43]. To investigate the capability of RVG29/TK@Lip/GYY in anti-nitrification stress, the levels of nNOS, nitrite and Nitro-α-syn were determined, respectively. As depicted in Fig. 5G, nNOS expression in the SN and ST of PD model mice was 48.0% and 66.6% higher than that of the Ctrl group, respectively. By contrast, treatment with RVG29/TK@Lip/GYY significantly decreased nNOS levels by 33.2% in the SN and 41.6% in the ST, respectively, compared with the PD model group (Fig. 5H and S12). Besides, nitrite measurements revealed that RVG29/TK@Lip/GYY significantly reduced the level of nitrite in the ST (Fig. 5I). Moreover, the IHC staining results of Nitro-α-syn in above groups suggested that PD model group displayed a more Nitro-α-syn proportion in both SN and ST than that of Ctrl group (Fig. 5J). However, RVG29/TK@Lip/GYY treatment effectively reduced the density and content of Nitro-α-syn in comparison with other treatment groups. These findings hinted that RVG29/TK@Lip/GYY is capable of efficaciously relieving nitrative stress in PD brain.

### 2.8 RVG29/TK@Lip/GYY alleviates neuroinflammation and restores microglial homeostasis

Given the potential effect of H_2_S in regulating inflammatory response, we subsequently evaluated the therapeutic efficacy of RVG29/TK@Lip/GYY in improving microglial function and ameliorating neuroinflammation. Firstly, the activation states of microglia and astrocytes in each group were assessed by IHC staining using ionized calcium binding adaptor molecule 1 (Iba-1, a microglial activation marker) and glial fibrillary acidic protein (GFAP, an astroglial activation marker), respectively. As depicted in Fig. 6A and B, compared with the Ctrl group, PD model mice exhibited a significant increase in the number of Iba-1^+^ microglia and GFAP^+^ astrocytes in the ST and SN. Additionally, these cells displayed an amoeboid morphology characterized by enlarged cell bodies and thickened processes, indicating excessive microglial and astroglial activation in the PD brain. On the contrary, RVG29/TK@Lip/GYY-treated group displayed a branched morphology with small cell bodies, which was similar to the Ctrl group mice, suggesting the mitigation of microglial and astrocyte activation of RVG29/TK@Lip/GYY. Next, WB results of CD16/32 (M1-like pro-inflammatory activation) and CD206 (M2-like neuroprotective phenotype) in the ST of each group indicated that the PD model group exhibited a 56.1% decrease of CD206 vs CD16/32 ratio compared with the Ctrl group (Fig. 6C and D). However, the treatment of RVG29/TK@Lip/GYY could markedly increase the level of CD206 vs CD16/32 ratio in comparison with other treatment groups, suggesting RVG29/TK@Lip/GYY could effectively promote M2-like microglial polarization. Meanwhile, the levels of inflammatory cytokines in the ST of each group mice were also determined by qRT-PCR. As shown in Fig. 6E–J, the treatment of RVG29/TK@Lip/GYY effectively downregulated the expression of pro-inflammatory cytokines (TNF-α, IL-1β and IL-6) and concurrently upregulated the expression of anti-inflammatory cytokines (IL-10, Arg-1 and TGF-β), thereby suppressing excessive immune activation. In addition, the above phenotypic markers and pro/anti-inflammatory cytokines expression in the SN of RVG29/TK@Lip/GYY-treated PD mice also demonstrated a reduction in activated microglia (Figs. S13 and S14). Overall, these results implied that RVG29/TK@Lip/GYY can effectually ameliorate microglial activation and relieve neuroinflammation in PD.

**Fig. 6:**
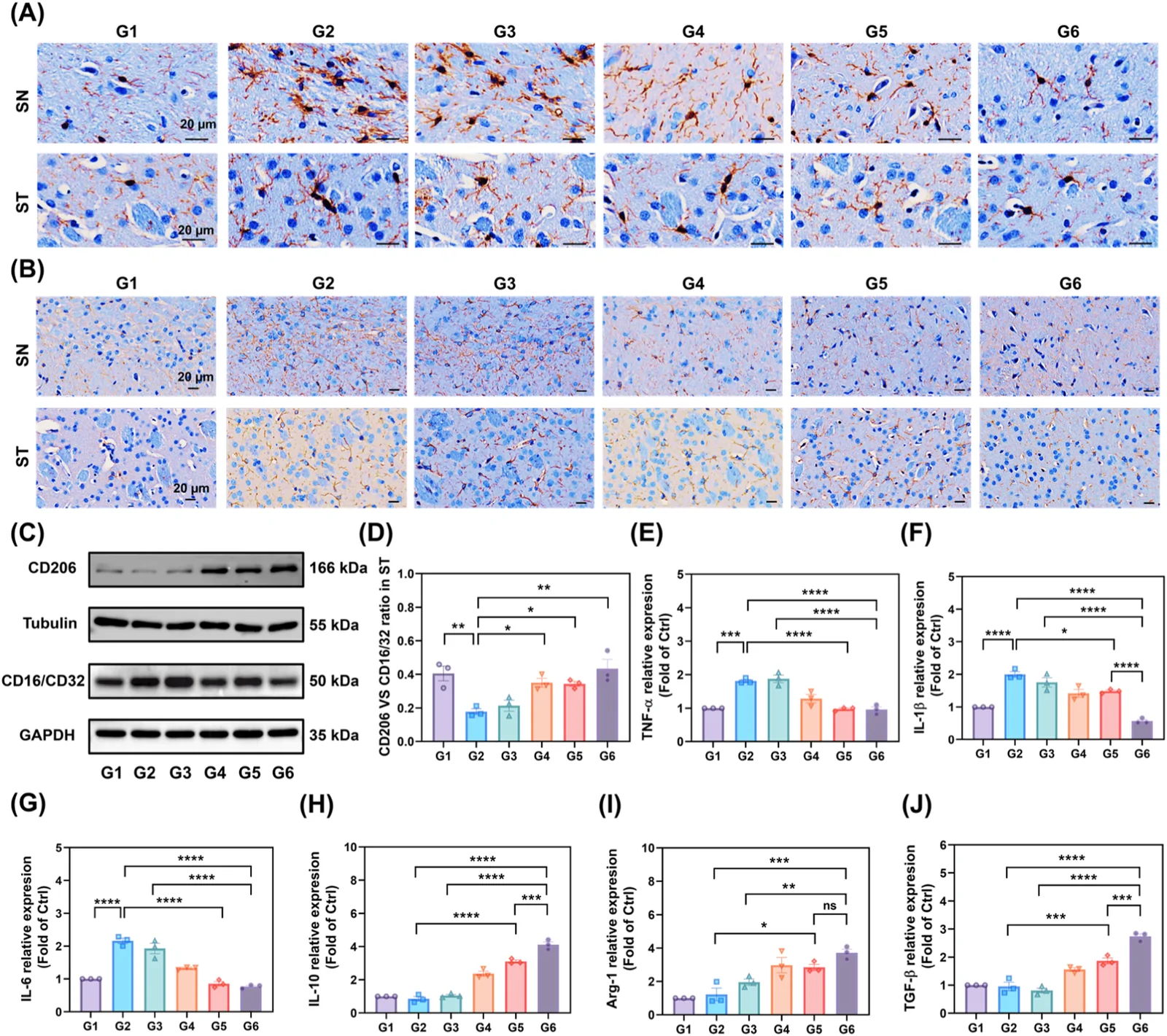
The capacities of RVG29/TK@Lip/GYY on alleviating neuroinflammation and restoring glial cells homeostasis. The expression of (A) activated microglia and (B) activated astrocyte in the SN and ST; scale bar = 20 μm. (C) WB assays of phenotypic markers after different formulations intervention in the ST. (D) The relative expression of CD206 VS CD16/32 from (C). The mRNA expression of (E) TNF-*α*, (F) IL-1*β*, (G) IL-6, (H) IL-10, (I) Arg-1, and (J) TGF-*β* in the ST. Results are reported as means ± SEM (*n* = 3); one-way ANOVA; TuKey's multiple comparisons test; *p < 0.05, **p < 0.01, ***p < 0.001, ****p < 0.0001 and ns, not significant.

Furthermore, H&E staining was performed to evaluate the biosafety of different treatment. Histological examination (Fig. S15) revealed no significant morphological differences between all treatment groups and the Ctrl group, indicating that RVG29/TK@Lip/GYY did not cause obvious organ toxicity. These results indicated the good biocompatibility and promising application of RVG29/TK@Lip/GYY in PD brain therapy.

## 3 Conclusion

In summary, to achieve the precise, efficient and controllable release of H_2_S in PD brain, we developed a H_2_S nanocarrier, RVG29/TK@Lip/GYY, that capable of brain-targeting, BBB-penetrating and ROS-responsive approach for effectively H_2_S delivery in PD treatment. Our results suggested that after crossing BBB, RVG29/TK@Lip/GYY can response the microenvironmental ROS in PD neural regions and enable to release H_2_S specifically. Importantly, *in vivo* results demonstrated that RVG29/TK@Lip/GYY could effectually improve the motor function of PD model mice greater extent than other component therapy methods. Mechanically, RVG29/TK@Lip/GYY efficiently mitigated oxidative and nitrative stress, alleviated neuroinflammation, and restored dopaminergic neurons integrity, thereby a conferring neuroprotective and anti-apoptotic effect. Taken together, this research promoted the feasibility of precise and controllable releasing H_2_S for PD intervention and also offered a technical foundation for gas therapy in other neurodegenerative disorders.

## CRediT authorship contribution statement

**Jia Ke:** Writing – original draft, Software, Methodology, Data curation. **Shiyi Yu:** Software, Methodology, Formal analysis, Data curation. **Hong Yuan:** Supervision, Project administration, Conceptualization. **Fuqiang Hu:** Writing – review & editing, Supervision, Funding acquisition, Conceptualization.

## Declaration of competing interests

The authors declare that they have no known competing financial interests or personal relationships that could have appeared to influence the work reported in this paper.

## Data Availability

Data will be made available on request.

## Appendix A Supplementary data

Additional information of materials, instruments, experimental procedures, ^1^H NMR characterization spectra and other experimental figures were provided in the supporting information.
