# Supplementary material for: Brain-penetrating and lesion-triggered hydrogen sulfide nanocarrier for multiple pathogenic interventions in Parkinson's disease therapy
Source: Mater Today Bio. 2026 Nov 3;41:103806. doi: 10.1016/j.mtbio.2026.103806 (PMC13769527; doi:10.1016/j.mtbio.2026.103806)
Supplement: Supplementary file 1 [file mmc1.docx]

**Supporting Information**

**Brain-penetrating and lesion-triggered hydrogen sulfide nanocarrier for multiple pathogenic interventions in Parkinson's disease therapy**

*Jia Ke*^a,b,1^*, Shiyi Yu*^b,1^*, Hong Yuan*^b,c^*, Fuqiang Hu* ^b,c^*^*^*

^a^ *Department of Pharmacy, Affiliated Hospital of Zunyi Medical University, Zunyi 563000, China*

^b^ *College of Pharmaceutical Science, Zhejiang University, Hangzhou 310058, China*

^c^ *National Key Laboratory of Advanced Drug Delivery and Release Systems, Zhejiang University, Hangzhou 310058, China*

^1^ *These authors equally contributed to this work: Jia Ke, Shiyi Yu*

* *Corresponding author: Fuqiang Hu, E-mail: hufq@zju.edu.cn.*

**1. Experimental Section**

***Materials***: Morpholin-4-ium (4-Methoxyphenyl) (morpholino) phosphorodithioate (GYY4137, SML0100) was purchased from Sigma-Aldrich (USA). 1,2-distearoyl-sn-glycero-3-phosphoethanol-amine-ketosulfone-N-[methoxy(polyethylene glycol)-2000] (DSPE-TK-PEG_2000_) was purchased from Xi'an Ruixi Biological Technology Co., Ltd (Xi 'an, China). 1,2-distearoyl-sn-glycero-3-phosphoethanol-amine N-[methoxy(polyethylene glycol)-2000] (DSPE-mPEG_2000_) and 1,2-distearoyl-sn-glycero-3-phosphoethanolamine-N-[maleimide(polyethylene glycol)-2000] (DSPE-PEG_2000_-MAL) were purchased from Shanghai Pengsheng Biological Technology Co., Ltd (Shanghai, China). RVG29 peptide with an additional cysteine on its C-terminal (RVG29-Cys) was synthesized by GL Biochemical Co., Ltd (Shanghai, China). The peptide sequence of RVG29-Cys is YTIWMPENPRPGTPCDIFTNSRGKRASNGC. Cholesterol was purchased from Sinopharm chemical reagent Co., Ltd (Shanghai, China). Distearoyl phosphatidylcholine (DSPC) was purchased from AVT(Shanghai) Pharmaceutical Tech Co. Ltd (Shanghai, China). H_2_S content assay Kit was purchased from Solarbio Technology Co., Ltd (BC2055, Beijing, China). Reactive Oxygen Species Assay Kit was purchased from Beyotime Biotechnology Co., Ltd (Shanghai, China). 3-(4,5-dimethylthiazol-2-yl)-2,5-diphenyltetrazolium bromide (MTT) and Hoechst 33342 were purchased from Sigma-Aldrich (USA). 1-methyl-4-phenylpyridinium (MPP^+^) iodide was purchased from Shanghai Macklin Biochemical Technology Co., Ltd (Shanghai, China). 1-methyl-4-phenyl-1,2,3,6-tetrahydropyridine (MPTP) hydrochloride was purchased from purchased from Sigma-Aldrich (USA).

***Cell lines***: The bEnd.3 and SH-SY5Y cell lines were obtained from the Chinese Academy of Sciences Cell Bank (Shanghai, China). For culture, bEnd.3 and SH-SY5Y cells were maintained in Dulbecco’s Modified Eagle’s Medium (DMEM; Gibco) enriched with 10% fetal bovine serum (FBS; Gibco). All cells were incubated in an incubator at 37 °C with 5% CO_2_ and 99% humidity.

***Animals***: C57BL/6 mice (male, 8-10 weeks) were purchased from the Hangzhou Qizhen Laboratory Animal Science and Technology Co., LTD (Hangzhou, China). The mice were group-housed and maintained in a 12-hour light-dark cycle within a temperature-controlled environment. All animal procedures were ethically approved by the Zhejiang University Animal Ethics Committee. The animal ethics approval number is ZJU20250127.

***Synthesis of DSPE-PEG_2000_-RVG***: DSPE-PEG_2000_-RVG29 was synthesized via a maleimide-thiol reaction between DSPE-PEG_2000_-MAL and RVG29-Cys. Briefly, RVG29-Cys was dissolved in dimethylformamide (DMF), and then DSPE-PEG_2000_-MAL was added to the mixture with a molar ratio of 1.4:1. The mixture was gently stirred overnight at room temperature in the dark. The resulting supernatant was dialyzed against distilled water for 48 h. The purified dialysate was lyophilized and stored at -20 °C.

***Preparation of RVG29/TK@Lip/GYY****:* Preparation of brain targeting H_2_S nanocarrier adopted the thin-film dispersion method. Briefly, DSPC (7.0 mg), cholesterol (2.0 mg), DSPE-TK-PEG_2000_ (1.0 mg), DSPE-mPEG_2000_-RVG29 (1.0 mg) and GYY4137 (1.0 mg) were accurately weighed and dissolved in a mixture of dichloromethane and methanol (3:1, v/v) to obtain a transparent solution. The organic solvent was evaporated by rotary evaporation for one night. After that, the film was hydrated with 11.0 mL of deionized water, and the solution was slowly stirred at 37 °C for 30 min. Then, it was ultrasonicated at 4 °C with a 400 W probe (40 cycles, 2 seconds on, 3 seconds off) to obtain the ROS-sensitive brain-targeted H_2_S nanocarrier RVG29/TK@Lip/GYY. By replacing 1.0 mg of DSPE-mPEG_2000_-RVG29 with 2.0 mg of DSPE-mPEG_2000_, the non-ROS-sensitive non-brain-targeted delivery system Lip/GYY was obtained.

***Characterization and stability evaluation***: TEM images were obtained JEM-1400 Flash (TEM, JEOL Ltd., Japan). The particle size and zeta potential were recorded by a Zetasizer (Nano-S90, Malvern, UK). ^1^H nuclear magnetic resonance (^1^H NMR) spectra were recorded on a Bruker AVIII500M spectrometer (Germany). The stability of RVG29/TK@Lip/GYY dispersed in 10% FBS/PBS (v/v) was evaluated by measuring particle size changes during storage at 4 °C for 5 days.

***Encapsulation efficiency and drug loading of RVG29/TK@Lip/GYY***: The encapsulation efficiency (EE, %) and drug loading (DL, %) of GYY4137 were determined using the ultrafiltration-centrifugation method. Specifically, RVG29/TK@Lip/GYY and Lip/GYY solutions were placed in ultrafiltration centrifugation tubes (10 kDa) and centrifuged (3166×g, 30 min) to separate free GYY4137. The concentration of free GYY4137 was measured in triplicate with a H_2_S detection kit. EE% and DL% were calculated by using the following equations, respectively.

EE (%) = $\frac{M_{\mathrm{GYY}}-C_{\mathrm{Free}} \times V}{M_{\mathrm{GYY}}}\times100\%$

DL (%) = $\frac{M_{\mathrm{GYY}}-C_{\mathrm{Free}}\times V}{M_{\mathrm{GYY}}+M_{\mathrm{liposome}}}\times100\%$

Where the *M*_GYY_ represents the weight of total GYY4137, and *C*_free_ is the GYY4137 concentration in the filtrate of the ultrafiltration centrifuge tube, *V* is the volume of the filtrate from the ultrafiltration centrifuge tube, *M*_liposome_ is the liposome weight.

***In vitro ROS-sensitive release analysis***: The dialysis method was performed the in vitro release of GYY4137. Briefly, 1 mg/mL of RVG29/TK@Lip/GYY and Lip/GYY solution were suspended in a dialysis bag (MWCO = 3.5 kDa) and incubated in 10 mL of phosphate buffer solutions (PBS, pH 7.4) with or without H_2_O_2_ (100 μM). The system was shaken in a water bath at 37 °C with stirring at 100 rpm. At predetermined time intervals, all the release medium was taken and replaced with fresh medium at the same temperature. The concentration of GYY4137 as determined using the H_2_S kit, and the cumulative release percentage of GYY4137 was calculated.

***Cytotoxicity assay***: The biocompatibility of H_2_S nanocarrier was evaluated by MTT assay. Generally, the SH-SY5Y cells were seeded on a 96-well plate at a density of 7×10^3^ cells/well and allowed to attach overnight. Then, the culture medium was replaced by a serum-free medium containing RVG29/TK@Lip/GYY and Lip/GYY for 24 h. After that, 20 μL MTT solution (5 mg/mL) was added to the wells and incubated for another 4 h. Then the solution in the well was aspirated gently and 200 μL DMSO was added into each well to dissolve the formazan crystal. The absorbance was measured at a wavelength of 570 nm. The cell viability (%) was calculated by the following formula:

Cell viability (%) = $\frac{A_{1}}{A_{2}}\times100\%$

where the *A*_1_ and *A*_2_ represent the OD values of treatment group, and control group, respectively.

***Cellular uptake analysis***: RVG29/TK@Lip/DiI and Lip/DiI were prepared at equivalent total lipid concentrations (on a mass basis) through encasing the same amount of a red fluorescent probe DiI*.* In detail, DSPC (7.0 mg), cholesterol (2.0 mg), DSPE-TK-PEG_2000_ (1.0 mg), DSPE-mPEG_2000_-RVG29 (1.0 mg) and DiI (1.0 mg) were accurately weighed and dissolved in a mixture of dichloromethane and methanol (3:1, v/v) to obtain a homogeneous solution. The organic solvent was evaporated by rotary evaporation for one night. After that, the film was hydrated with 11.0 mL of deionized water, and the solution was slowly stirred at 37 °C for 30 min. Then, it was ultra-sonicated at 4 °C with a 400 W probe (40 cycles, 2 seconds on, 3 seconds off) to obtain RVG29/TK@Lip/DiI. Lip/DiI was prepared by using DSPC (7.0 mg), cholesterol (2.0 mg), DSPE-mPEG_2000_ (2.0 mg) and DiI (1.0 mg).

Then, bEnd.3 and SH-SY5Y cells were seeded on 24-well plates at 10×10^4^ cells/well for 12 h. The medium was then removed and replaced with fresh culture medium containing RVG29/TK@Lip/DiI and Lip/DiI, which containing 10 μM DiI. After 1 h and 4 h incubation, followed by staining with Hoechst (250 nM) for 5min. Cellular uptake of RVG29/TK@Lip/DiI and Lip/DiI were observed with laser scanning confocal microscopy (CLSM, IX81-FV1000, Olympus, Japan).

For quantitative analysis of cellular uptake, SH-SY5Y cells were seeded at a density of 20×10^4^ cells/well in 6-well plates and cultured at 37 °C for 24 h. Then RVG29/TK@Lip/DiI and Lip/DiI, which containing 10 μM DiI, were incubated with cells for another 4 h, the cells were washed three times with PBS. The single-cell suspension was prepared and quantified by flow cytometry (Cytoflex, Beckman Coulter, USA).

***In vitro BBB model transcytosis***: The transwell experiment was used to investigate the capability of RVG29/TK@Lip and Lip to penetrate BBB. *In vitro* BBB models was firstly established, bEnd.3 cells were seeded in the upper chambers (pore size: 0.4 μm, Corning) at 1×10^5^ cells/insert. During the culture for 14 days, the integrity of cell monolayer was verified by measuring the transepithelial electrical resistances (TEER > 150 Ω·cm^2^) values using a Millicell-ERS volt-ohmmeter (Millipore Co., USA). After that, SH-SY5Y cells (1×10^5^ cells/insert) were seeded on the lower chamber and co-incubation for 24 h. Then the criterion of TEER ≥ 150 Ω·cm^2^ as indicative of intact barrier integrity, suggesting the success of establishing *in vitro* BBB model. RVG29/TK@Lip/DiR and Lip/DiR with 100 μM DiR were added into upper inserts. After 4 h incubation, SH-SY5Y cells in the lower chamber were rinsed and collected in PBS. Then the cells were assessed with flow cytometry (Cytoflex, Beckman Coulter, USA) to determine the fluorescence intensity of DiR. All samples were tested in triplicates.

***Determination of intracellular ROS***: SH-SY5Y cells were seeded at a density of 1×10^4^ cells/well in a confocal dish and cultured for 24 hours. Then RVG29/TK@Lip, GYY4137, Lip/GYY and RVG29/TK@Lip/GYY (containing 100 μM GYY4137) were added and continued incubation for pre-intervening 1 h. Then 500 μM MPP^+^ were added to the confocal dish and incubated for 24 hours. After incubation, PBS was used to gently wash cells, and then successively added DCFH-DA (200 μL, 10 μM) for 30 min, CLSM was used to observe ROS fluorescence images in SH-SY5Y cells. ***Investigation of anti-nitrification stress***: The anti-nitrification stress of RVG29/TK@Lip/GYY was evaluated by western blot (WB). SH-SY5Y cells were seeded in 6-well plate at a density of 20×10^4^ cells/well and incubated until 90% confluence was achieved. Cells were treated as above mentioned and then collected by scraping and lysed with RIPA lysis solution. Protein concentration was determined with Bradford protein quantification kit. Totally, 30 μg proteins were separated by 8% SDS-PAGE and transferred onto PVDF membranes. The membranes were blocked with 5% skim milk and incubated with anti-nNOS (1:500, R1510-28, HUABIO Biotechnology Co., Ltd, China), and anti-*β*-actin (1:3000, 81115-1-RR, Wuhan Sanying Biotechnology Co., Ltd, China) at 4 ℃ overnight, followed by HRP-conjugated secondary antibody at room temperature for 1 h. Bands were detected by ECL reagents and analyzed by Image Lab (Bio-Rad) with *β*-actin normalization.

***Apoptosis assay***: The Annexin V-FITC/PI apoptosis detection kit (Dalian Meilun Biotech Co., China) was used to evaluate the SH-SY5Y cells apoptosis according to the instructions. Briefly, SH-SY5Y cells were seeded at a density of 5×10^4^ cells/well in 12-well plate and incubated overnight. Then RVG29/TK@Lip, GYY4137, Lip/GYY and RVG29/TK@Lip/GYY (containing 100 μM GYY4137) were added and continued incubation for pre-intervening 1 h. Then 500 μM MPP^+^ were added to the confocal dish and incubated for 24 h. Then the single-cell suspension was prepared, and the percentage of apoptosis was determined via flow cytometry (FCM). Cell debris was first excluded by FSC-A/SSC-A gating, and doublets were excluded by FSC-A/FSC-H gating. Single cells were then analyzed on the Annexin V FITC versus PI dot plot, with quadrant gates set based on unstained and single-stained controls.

***Establishing PD animal model***: The PD model was induced in C57BL/6 mice (male, 8-10 weeks) according to a common regimen, which involves one injection of MPTP (16 mg/kg) every 2 h for a total of four doses over an 8 h period in 1 d. All animal experiments were bred and maintained in a specific pathogen free barrier facility, and all procedures were approved and performed in accordance with national guidelines of the ethical committee of Zhejiang University (ZJU20250127).

***Intracerebral delivery efficiency and distribution***: The RVG29/TK@Lip and Lip were firstly labeled with DiR to obtain RVG29/TK@Lip/DiR and Lip/DiR, which were subsequently injected into PD mice through tail vein. The fluorescence signals were recorded at different times using the Maestro EX 2.10 *in vivo* Imaging System (CRI, Woburn, USA). Additionally, the brains and main organs (heart, liver, spleen, lung, kidney) of mice in each group were isolated for observation at the 4 h. The fluorescence signals of DiR signals were recorded and analyzed.

***Animal administration procedure***: mice were randomly divided into six groups (*n* = 6):

Group 1 (G1): Ctrl, wild-type mice.

Group 2 (G2): PD model, mice were intraperitoneal injection with MPTP (16 mg/kg), injection every two hours, four times in total.

Prior to modeling, mice were tail vein injection with RVG29/TK@Lip as Group 3 (G3), with GYY as Group 4 (G4), with Lip/GYY as Group 5 (G5), and with RVG29/TK@Lip/GYY as Group 6 (G6) for continuous three days, once a day, and the dosage was 130 μM/kg according to GYY content. Each group mice were then intraperitoneal injection with MPTP (16 mg/kg) four times, once two hours. After modeling, the mice in each group were treated with different formulations for another 7 days.

***Behavioral experiments***: After treatment as above described, motor function of mice was evaluated by the open field test, pole-climbing test and rotarod test, respectively.

***The open field test***: To evaluate the autonomous movement behavior and anxiety-like behavior of PD mice after treatment, the open field test was performed as described. Each mouse adapted to the open field test box (45 cm × 45 cm × 45 cm) for 3 days before formal test. The mice freely explored for 5 minutes, then the movement trajectory, total movement distance, movement distance in the central area, total rest time and average speed of the mice were recorded by Any-maze software.

***Pole-climbing test***: The pole-climbing test was performed to evaluate the motor coordination ability of PD mice after treatment. Each mouse underwent adaptive training for 3 days, with 3 training sessions per day. During the test procedure, the mice were placed positioned at the apex of the pole, the mice were guided to turn their heads and climb from the top to the bottom along the apparatus.

***Rotarod test***: The rotarod test was carried out to evaluate the motor coordination ability and balance ability of PD mice after treatment. Each mouse underwent adaptive training for 3 days, with 3 training sessions per day. Mice were placed on a rotating rod set to accelerate from 4 to 40 rpm/min, and the latency to fall off was automatically recorded by the device.

***H_2_S and NO levels measurement***: After treatment, dissected brain tissues were rinsed with PBS (pH 7.4) and lysed with extracting solution, supernatants were collected by centrifugation at 12,000 g for 10 min. The content detection kit was used to determine the concentration of H_2_S and NO according to the manufacturer's protocols.

***Immunohistological staining study***: After treatment as described above, the brain and major organs (heart, liver, spleen, lung, and kidney) of mice were removed to perform immunohistological staining. The tissue samples were fixed with 4% PFA for 24 h, dehydrated, impregnated with wax, embedded, sectioned, stained, and sealed. Tissue sections were stained using TH, Iba1, GFAP and hematoxylin and eosin (H&E).

***Statistical analysis***: All statistical analyses were performed using GraphPad Prism software. Data are presented as mean ± standard error of the mean (SEM). The diﬀerence between the two groups was assessed by using an unpaired t-test. For multiple comparisons, one-way analysis of variance was used to analyze the statistical significance. Representation of the *p*-value was **p* < 0.05, ***p* < 0.01, ****p* < 0.001, *****p* < 0.0001 and ns, not significant.

**2. Supplementary figures**

**Figure S1**. (A) Synthesis route DSPE-PEG_2000_-RVG29. (B) ^1^H NMR spectra of DSPE-PEG_2000_-MAL, RVG29-Cys and DSPE-PEG_2000_-RVG29 in DMSO-*d*_6_.

**Figure S2**. Stability of RVG29/TK@Lip/GYY at serum environment (10% FBS). Results are reported as means ± SEM (*n* = 3).

**Figure S3**. Change of volume-weighted size distribution of Lip/GYY in the high ROS microenvironment over time (*n* = 3).

**Table S1**. The volume-weighted size and its distribution percentage of RVG29/TK@Lip/GYY in the high ROS microenvironment.

**Figure S4**. The mechanistic process of TK cleavage from DSPE-TK-PEG_2000_ by ROS.

**Figure S5.** Chemical structures of GYY4137 and the proposed mechanism for H_2_S release from GYY4137.

**Figure S6**. The cell viability of SH-SH5Y cells treated with (A) GYY and (B) Lip/GYY. Results are reported as means ± SEM (*n* = 3); one-way ANOVA; TuKey's multiple comparisons test; **p* < 0.05, ***p* < 0.01, ****p* < 0.001, *****p* < 0.0001 and ns, not significant.

**Figure S7**. Total apoptosis ratio statistics of Fig. 3F. Results are reported as means ± SEM (*n* = 3); one-way ANOVA; TuKey's multiple comparisons test; **p* < 0.05, ***p* < 0.01, ****p* < 0.001, *****p* < 0.0001 and ns, not significant.

**Figure S8**. Cell viability of SH-SY5Y cells of different treatment groups. Results are reported as means ± SEM (*n* = 3); one-way ANOVA; TuKey's multiple comparisons test; *****p* < 0.0001.

**Figure S9.** Quantitative fluorescent intensity analysis of images in Fig. 4B.

**Figure S10**. (A) The fluorescence image of main organs after intravenously injection different formulations at 4 h. (B) The fluorescence intensity of Lip/DiR and RVG29/TK@Lip/DiR in the brain tissues at 4 h from (A). Results are reported as means ± SEM (*n* = 3); one-way ANOVA; TuKey's multiple comparisons test; **p* < 0.05, ***p* < 0.01, ****p* < 0.001, *****p* < 0.0001 and ns, not significant.

**Figure S11**. Semi-quantitative analysis of TH expression in the SN (A) and ST (B) of different treatment groups from Fig. 5D. Results are reported as means ± SEM (*n* = 3); one-way ANOVA; TuKey's multiple comparisons test; **p* < 0.05, ***p* < 0.01, ****p* < 0.001, *****p* < 0.0001 and ns, not significant.

**Figure S12**. Semi-quantitative analysis of nNOS expression in the ST of different treatment groups from Fig. 5G. Results are reported as means ± SEM (*n* = 3); one-way ANOVA; TuKey's multiple comparisons test; **p* < 0.05, ***p* < 0.01, ****p* < 0.001, *****p* < 0.0001 and ns, not significant.

**Figure S13**. (A) Western blot assays of phenotypic markers in the SN after different formulations intervention. (B) The relative expression of CD206 VS CD16/32 in the SN. Results are reported as means ± SEM (*n* = 3); one-way ANOVA; TuKey's multiple comparisons test; **p* < 0.05, ***p* < 0.01, ****p* < 0.001, *****p* < 0.0001 and ns, not significant.

**Figure S14**. (A-F) The mRNA expression of TNF-*α*, IL-1*β*, IL-6, IL-10, Arg-1 and TGF-*β* in the SN were determined by qRT-PCR. Results are reported as means ± SEM (*n* = 3); one-way ANOVA; TuKey's multiple comparisons test; **p* < 0.05, ***p* < 0.01, ****p* < 0.001, *****p* < 0.0001 and ns, not significant.

**Figure S15**. H&E staining of various groups mice after different formulations treatment.
